# A finite element study of the effect of cross-link stabilisation in a lumbar spine tumour model

**DOI:** 10.1177/09544119251348279

**Published:** 2025-07-07

**Authors:** Juntong Lai, James Tomlinson, Lee Breakwell, Damien Lacroix

**Affiliations:** 1 School of Mechanical, Aerospace and Civil Engineering, University of Sheffield, Sheffield, UK; 2 Insigneo Institute for Insilico Medicine, University of Sheffield, Sheffield, UK; 3 Sheffield Teaching Hospital NHS Foundation Trust, Sheffield, UK

**Keywords:** Finite element [Biomechanics], spine biomechanics, fixation, tumour, stabilisation

## Abstract

Spinal metastases can increase the risks of vertebral fracture due to bony destruction and instability in the spine. There are concerns that cross-links may impair adjuvant treatments, such as radiotherapy and proton beam therapy. The aim of this study was to assess the biomechanical effects of cross-link stabilisation for a growing tumour in order to provide recommendations on the use and placement of the cross-link. A finite element (FE) model of a fixation device was developed. The device was inserted virtually into a FE model of the lumbar spine (L1–S1) between L2 and L4. Tumour deposit of either 1.3%, 10.1%, 38.3%, 71.5% and 92.1% of the vertebral body was simulated. A 1000 N compressive, a 10° lateral bending and a 7.5 Nm torsional load were simulated on the top of L1. Results indicate that the stabilisation is capable of reducing the stress of the L3 lumbar spine under torsion with a growing tumour. However, compressive loading is concentrated in the L3 anterior vertebra when the tumour volume was greater than 10.1% of the vertebra volume. The cross-link stabilisation reduced the stress of the posterior body within the stabilised segments (L2–L4), especially under torsion. The position of the cross-link does affect the ability of stabilisation to reduce concentrated stress of both vertebrae and screws, which indicates that the position of the cross-link should be considered in clinical surgery to refine the stress concentration, spinal stability and structural stiffness, without compromising adjuvant treatments.

## Graphical abstract

**Graphical abstract:**
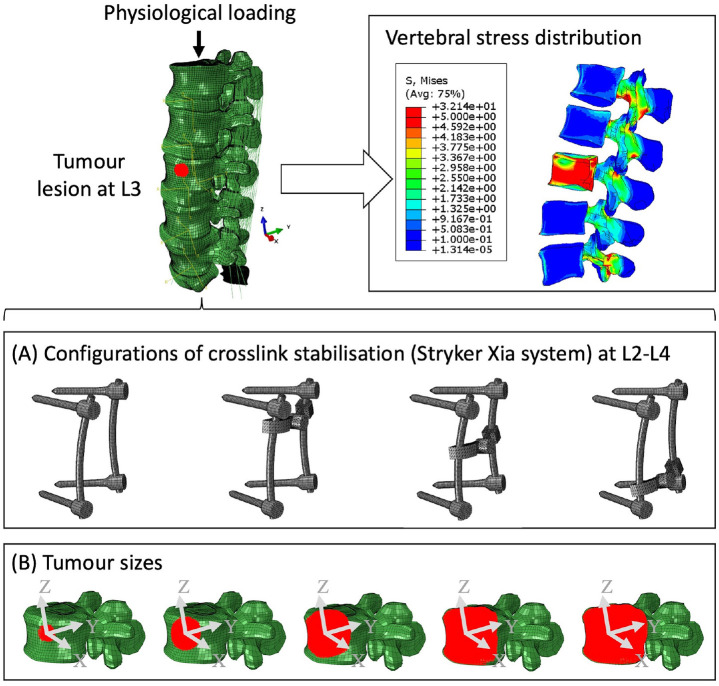

## Introduction

Despite the relatively rare occurrences of primary bone tumours in the lumbar spine,^1,2^ spinal metastases exist in nearly 70% of cancer cases.^3^ In general, stabilisation surgeries are indicated for neurological compression or instability.^4^ The construct allows relief of the spinal instability pain and stabilises the affected level of the spine. Cross links are known to increase the rigidity of the construct but may interfere with non-surgical therapies such as radiotherapy and proton beam therapy due to the titanium material of the cross-link partially disrupting the beam and reducing penetration at the tumour site.^5,6^ The risk of vertebral fracture significantly increases with the growing tumour lesion due to constant palliative treatments.^7,8^ Hence, it is necessary to assess the optimised condition of mounting a specific stabilisation system.

Surgery is normally dependent on the clinical assessment^9,10^ and factors such as tumour type (lytic or sclerotic) and the degree of bony destruction.^11^ Posterior stabilisation is the most common surgery for the decompression and stabilisation within a lumbar spine.^12–14^ Nevertheless, observable clinical performance and data after posterior stabilisation are not consistently precise and reliable for the quantitative assessment of spine biomechanics. Spinal surgery is commonly undertaken in patients with metastatic spine disease, usually due to instability pain, neurological compression or both. There is clear evidence that surgery combined with oncological treatment gives better outcomes in terms of pain, function and life expectancy than oncological treatment alone in this setting.^15^

The Finite Element (FE) method is well suited for the quantitative study of spine biomechanics.^16–21^ Cross-link stabilisation was shown to provide extra rotational stability,^13,22–26^ but may intensify the stress concentration in adjacent vertebrae due to changes in physiological load transfer.^27–30^ The position of the cross link may play a key role in reducing stress in stabilised vertebrae^31,32^ due to the asymmetrical motions of facet joints at different spinal levels.^33^ To the authors’ knowledge, there are no sensitivity studies of the impact of cross-link position within a metastatic lumbar spine. In addition, the effects of tumours on vertebral stress were evaluated from various perspectives including tumour volume, the location of tumour and tumour shape,^34,35^ and the nontrivial role of the tumour size in vertebrae was also consistent with clinical consensus.^36^ Nevertheless, it remains unclear how the tumour size and cross-linked positions of the stabilisation affect the biomechanics of the lumbar spine.

The main objectives of this study were twofold: (1) to analyse the effects of the tumour size on the intact and stabilised lumbar spines using FE models and (2) to analyse the impact of cross link position by shifting the position of cross-link within a specified lumbar spine tumour model.

## Methods

### Modification of FE models

Both patient-specific (CT and MRI data) and generic inputs in terms of geometry, material properties or boundary conditions were integrated within a complete L1–S1 FE model.^37–39^ Data was acquired as part of the EU-funded MySpine project by the National Center for Spinal Disorders (Budapest, Hungary) according to the national guidelines and institutional protocols. The clinical study was approved by the Scientific and Research Ethics Committee of the Hungarian Medical Research Council (751/PI/2010). The female patient (MY0216) of 76-year-old had disc degeneration at the L4–L5 level with a Pfirrmann grade of 3. The L3–L4 disc had a Pfirrmann grade of 2 and no bulging.

The intact patient-specific model includes 3325 linear line elements of type T3D2 for ligaments, 210,290 linear hexahedral elements of type C3D8 for bone, 71,472 linear hexahedral elements of type C3D8PH for intervertebral discs except caps, 25,920 linear hexahedral elements of type C3D8P for intervertebral disc caps, and 37,528 linear wedge elements of type C3D6 for sacrum. The total number of elements is 348,534 and the number of nodes is 348,690. The FE models were modified and implemented in ABAQUS 2021 (Dassault Systèmes, Vélizy-Villacoublay, France).

**Figure 1.**
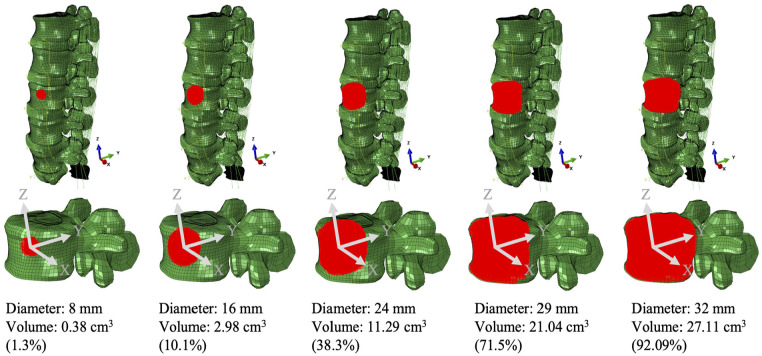
The tumour set within the L3 anterior vertebra (volume of 29.4 cm^3^).

Five tumour sets with volumes from 0.38 to 27.11 cm^3^ (Figure 1) were created within the L3 vertebra. The centroid of the tumour lesion was consistently located in the centre of the L3 anterior vertebra. The vertebra was not remeshed, instead the elements of the L3 anterior vertebra were selectively assigned with tumour properties giving an irregular shape close to a sphere for all tumour sizes.

**Figure 2.**
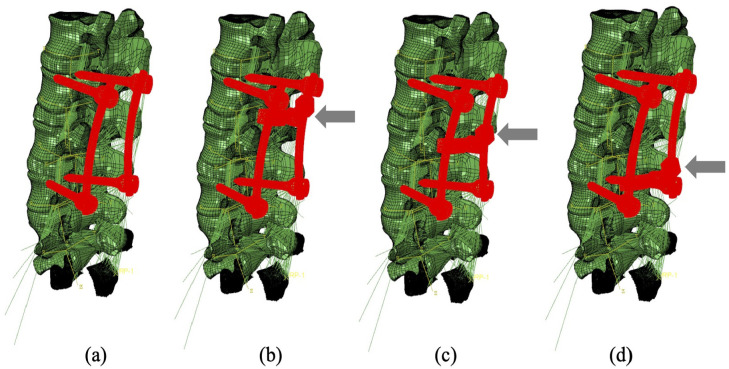
Four configurations of the fixation device: (a) device without cross-link bar, (b) device with the cross-link bar at the top, (c) device with the cross-link bar in the middle and (d) device with the cross-link bar at the bottom.

The cross-link stabilisation is the standard measurement of the Stryker implant (Stryker Xia system), including four pedicle screws (5 mm in diameter) with or without a cross-link bar which was positioned in three positions along the longitudinal rods (Figure 2). The bony elements, ligaments of overlap between the medical device and the tissues were removed to simulate orthopaedic practice.

### Material properties

Material properties used for the FE models are presented in Table 1 according to the anatomy of the functional spinal unit (FSU) in Figures S1–S3. The cortical bone and bony endplates were represented by a structural mesh layer on the outside of the vertebral body models. The thickness of this layer varied depending on the location, and local thickness values were defined from direct measurements on histological cuts.^40^ The material properties of cortical bone were anisotropic with 12,000 MPa in the axial direction and 8000 MPa in the transversal plane of cancellous bone.^41–43^ Personalisation of trabecular bone material properties is done with an element-specific approach in which each element of the trabecular region is assigned a specific Young’s modulus and Poisson ratio depending on the attenuation information contained in terms of grey values in each voxel building up the Computer Tomograph (CT). These values are translated into voxel-specific and then element-specific vascular porosity values. The vascular porosity enters a continuum micromechanics model for bone,^44^ which thereupon delivers voxel-specific properties for bone, as orthotropic material (Figure S3).

**Table 1.** Material properties of the components within the lumbar spine model.

Material	Young’s modulus (MPa)	Shear modulus (MPa)	Poisson’s ratio	Void ratio	Permeability (mm^4^/Ns)
Bony endplate^42^	1000		0.3		
Cortical bone^41–43^	8000	2000	0.4		
	8000	2400	0.35		
	12,000	2400	0.3		
Posterior elements^42^	3500		0.3		
Facet cartilage^42^	24		0.4		
Cartilage endplate^49^	20		0.1	4	7 × 10^−3^
Nucleus pulposus^49^	1.5		0.1	4.88	3 × 10^−4^
Anterior longitudinal ligament^47,48^	20		0.3		
Posterior longitudinal ligament^47,48^	20		0.3		
Intertransverse ligament^47,48^	58.7		0.3		
Ligamentum flavum^47,48^	19.5		0.3		
Capsular ligament^47,48^	32.9		0.3		
Supraspinous ligament^47,48^	15		0.3		
Interspinous ligament^47,48^	11.6		0.3		
Tumour lesion^46^	35	9.5	0.45		
	35	19.25	0.315	4	3 × 10^−3^
	62.5	19.25	0.3		
Titanium^5,23^	116,000		0.32		
Anisotropic hyperelastic material	The form of strain energy potential			Void ratio	Permeability (mm^4^/Ns)
Annulus fibrosus^48–50^	Holzapfel (*C*_10_ = 0.85, *K*_1_ = 2.8, *K*_2_ = 90)			3.55	3 × 10^−4^

Due to the tumour originating from the vertebral elements, orthotropic properties were determined for the tumour lesion^45^ with permeability.^46^ With respect to the ligaments, they were only considered to be acting in tension, and linear elastic properties were identified for all ligaments.^47,48^ Intervertebral discs were modelled as three main components (Figure S2): (1) an incompressible substance comprised of collagen fibrosus and isotropic materials including (2) cartilage endplate and (3) nucleus.^49^ Two families of fibres oriented at ±30° are continuously simulated using Holzapfel–Gasser–Ogden function,^50^ and are embedded within the nonlinear hyperelastic matrix of the annulus fibrosus.

The lumbar spine model with porous intervertebral disc and tumour was modified by creating a predefined field that provided the void ratio for the simulation of permeability within the intervertebral and tumour elements. Specifically, the predefined void ratio of 3.55 was determined for the annulus fibrosus,^48–50^ and the tumour^46^ was provided with the void ratio of 4 (see Table 1). In addition, the permeability was calculated as follows:

(1) $$k=k_{0}\;{\left[\frac{e\left(1+e_{0}\right)}{e_{0}\left(1+e\right)}\right]}^{2}\exp\left[8.5\left(\frac{1+e}{1+e_{0}}-1\right)\right]$$

where $k_{0}$ is the predefined permeability, and $e_{0}$ is the predefined void ratio.^41^

### Boundary conditions

The standard surface-to-surface contact was selected as the discretisation method to improve the convergence performance, moreover, penalty formulation with the coefficient of 0.005 was employed for the frictional behaviour due to the low friction characteristics of the synovia^51^ surrounding the facet joint. The embedded region was employed without friction for the constraints of the approximated rigid fixation device mounted to bony elements. The fixation device consists of four pedicle screws, two connecting rods and one cross-link bar.

Three loading conditions were simulated to the top endplate of the L1 vertebra: (1) a compressive force of 1000 N to simulate standing^21^; (2) a forward flexion of 10°^52^ and (3) a torsion of 7.5 Nm.^52^

## Results

### The effects of the stabilisation system and tumour size

**Figure 3.**
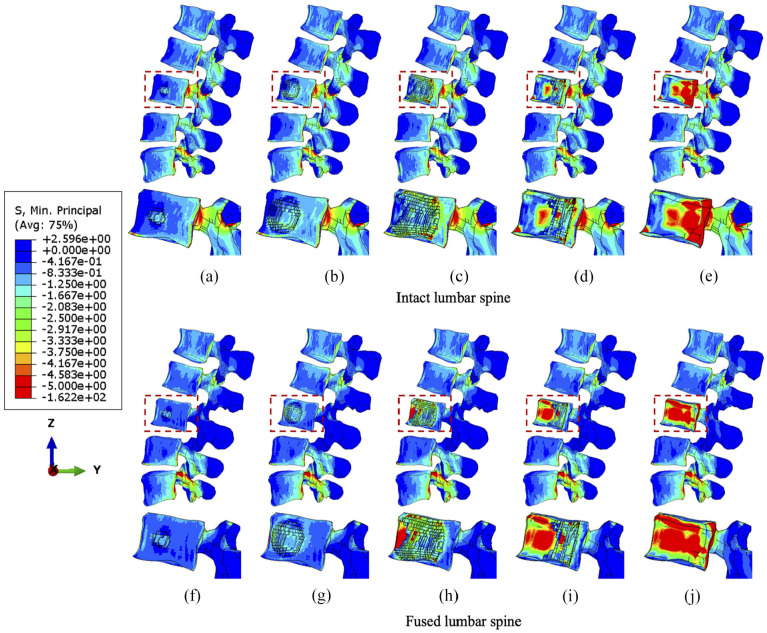
The minimum principal stress distribution of vertebrae with the incremental volumes of tumours under compression. Intact vertebrae with (a) 1.3%, (b) 10.1%, (c) 38.3 (d) 71.5% and (e) 92.1% tumour lesion. Fused vertebrae with (f) 1.3% (g) 10.1%, (h) 38.3%, (i) 71.5% and (j) 92.1% tumour lesion.

**Figure 4.**
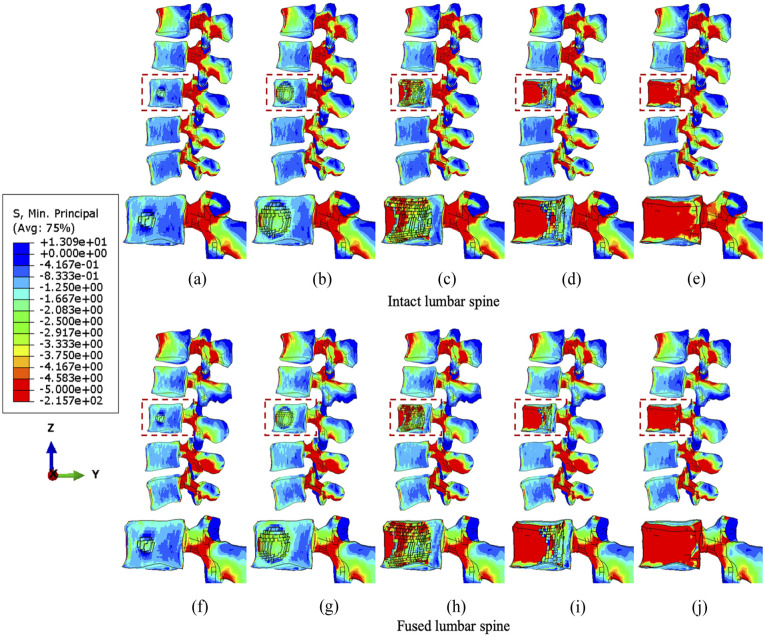
The minimum principal stress distribution of vertebrae with the incremental volumes of tumours under flexion. Intact vertebrae with (a) 1.3%, (b) 10.1%, (c) 38.3 (d) 71.5% and (e) 92.1% tumour lesion. Fused vertebrae with (f) 1.3% (g) 10.1%, (h) 38.3%, (i) 71.5% and (j) 92.1% tumour lesion.

**Figure 5.**
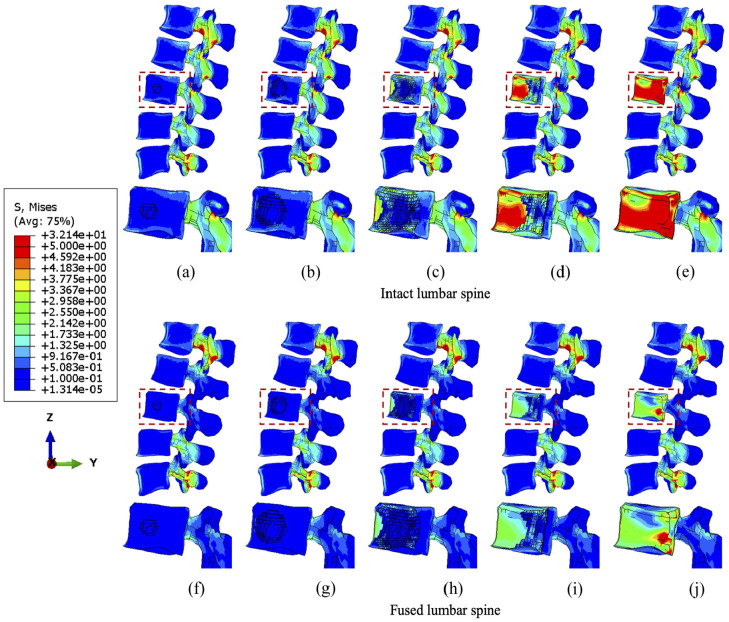
The von Mises stress distribution of vertebrae with the incremental volumes of tumours under torsion. Intact vertebrae with (a) 1.3%, (b) 10.1%, (c) 38.3 (d) 71.5% and (e) 92.1% tumour lesion. Fused vertebrae with (f) 1.3% (g) 10.1%, (h) 38.3%, (i) 71.5% and (j) 92.1% tumour lesion.

Results indicate that larger tumour sizes cause stress higher or more concentrated within the vertebrae (Figures 3–5). Figure 3 illustrates that the main vertebral body with tumour lesion is under higher compressive stress as well as the corresponding posterior regions. Although the pressure in the stabilised posterior elements is reduced, the stress of the L3 anterior vertebra is not significantly distributed when the tumour volume is more than 10.1% of the L3 vertebra in both intact and fused spines. Larger tumours (over 38.3%) stress is more concentrated in the edged elements of the L3 vertebra and transferred to the anterior edge from the pedicles after spinal stabilisation. Implants did not have a consistent positive impact on the stress reduction in the anterior vertebrae with increasing tumour volume. The compressive stress in the posterior elements is well reduced by the fixation device, but the stress concentration becomes much larger in the anterior elements with a larger tumour as the tumour causes erosion to most of L3 vertebral elements and the adjacent bone elements could resist less to compression.

Under flexion, the stress concentration around the tumour is not significantly increased until the tumour occupies 38.3% of the L3 vertebra (Figure 4). The fixation device does not result in significant differences in stress distribution to anterior vertebral body under flexion, but higher minimum principal stress is found in elements near the lesion due to the extra resistance provided by screws or rods embedded in the vertebrae. However, the posterior elements are under lower compressive stress when fused, which is consistent with the results under compression.

The von Mises stress distribution under torsion is less than 5 MPa in close proximity to the centroid of the anterior vertebrae as shear force is dominant and concentrated in posterior bony elements (Figure 5). Nevertheless, the relatively higher stress spreads to a larger number of elements near the tumour lesion from the edge of the L3 vertebra when the tumour volume increases to over 71.5%. Compared to compression and flexion, the stabilisation device could provide steady and continuous release of stress during torsion by elevating the resistance of the shear force within the fused segments, which persisted with incremental tumour sizes.

### Sensitivity study of cross-linked position

Computational data from the sensitivity study were visualised to contribute to optimising the position of a cross-link. Since larger tumour lesions are more likely to be stabilised, the lumbar spine models with over 71.5% tumour volume were selected to demonstrate the differences over four configurations of the cross-link stabilisation system. In particular, iso-surface is used for the visualisation of the specific regions under peak stress and the variations of vertebral stress distribution from the rear view, as the variations and gradient of stress distribution could be precisely captured in that case.

**Figure 6.**
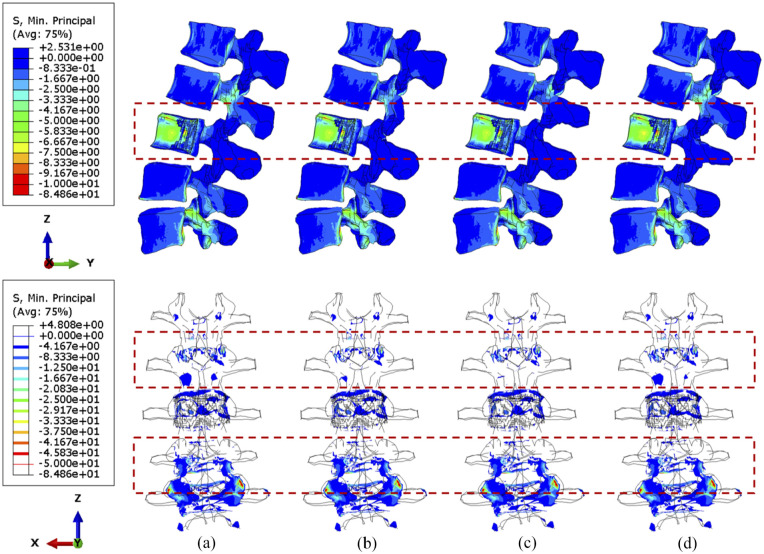
The minimum principal stress distribution of vertebrae, with 71.5% tumour volume, over different stabilisation system under compression. The stabilisation is configured: (a) without cross-link and with the cross-link, (b) at the top, (c) in the middle and (d) at the bottom.

**Figure 7.**
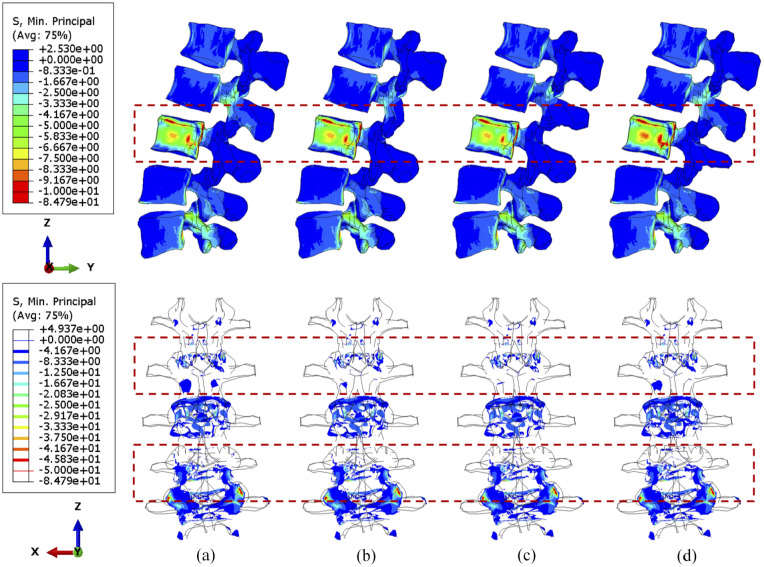
The minimum principal stress distribution of vertebrae, with 92.1% tumour volume, over different stabilisation systems under compression. The stabilisation is configured: (a) without cross-link and with the cross-link, (b) at the top, (c) in the middle and (d) at the bottom.

Under compression, the compressive stress (less than 5 MPa) is mainly concentrated to the L3 vertebra (Figure 6). In the side view, the stress distribution is not significantly different when shifting the cross-link position. Nevertheless, the number of posterior elements whose stress is less than 4 MPa decreased after stabilisation, which is highlighted in the upper adjacent vertebra in the rear view. Moreover, slightly less area of the L2/L3 facets are under high stress when the cross-link is mounted to the superior position, which can also be seen in Figure 7. A larger tumour lesion results in more intensive stress concentrated in the L3 anterior vertebra (comparison between Figures 6 and 7). Thus, the resultant stress release of the fixation device on the L3 anterior vertebra might be less effective when the tumour size is significantly large, as shown when the cross-link is located in the inferior part (Figure 7(d)).

**Figure 8.**
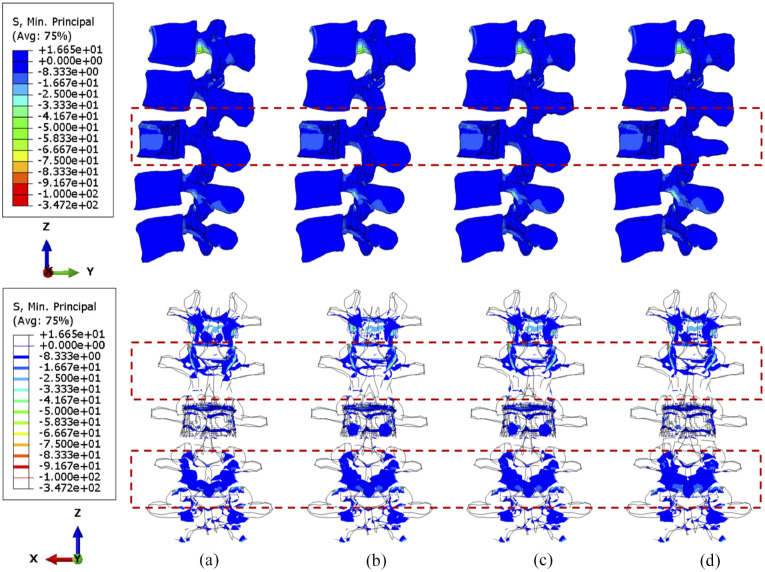
The minimum principal stress distribution of vertebrae, with 71.5% tumour volume, over different stabilisation systems while imposing flexion. The stabilisation is configured: (a) without cross-link and with the cross-link, (b) at the top, (c) in the middle and (d) at the bottom.

**Figure 9.**
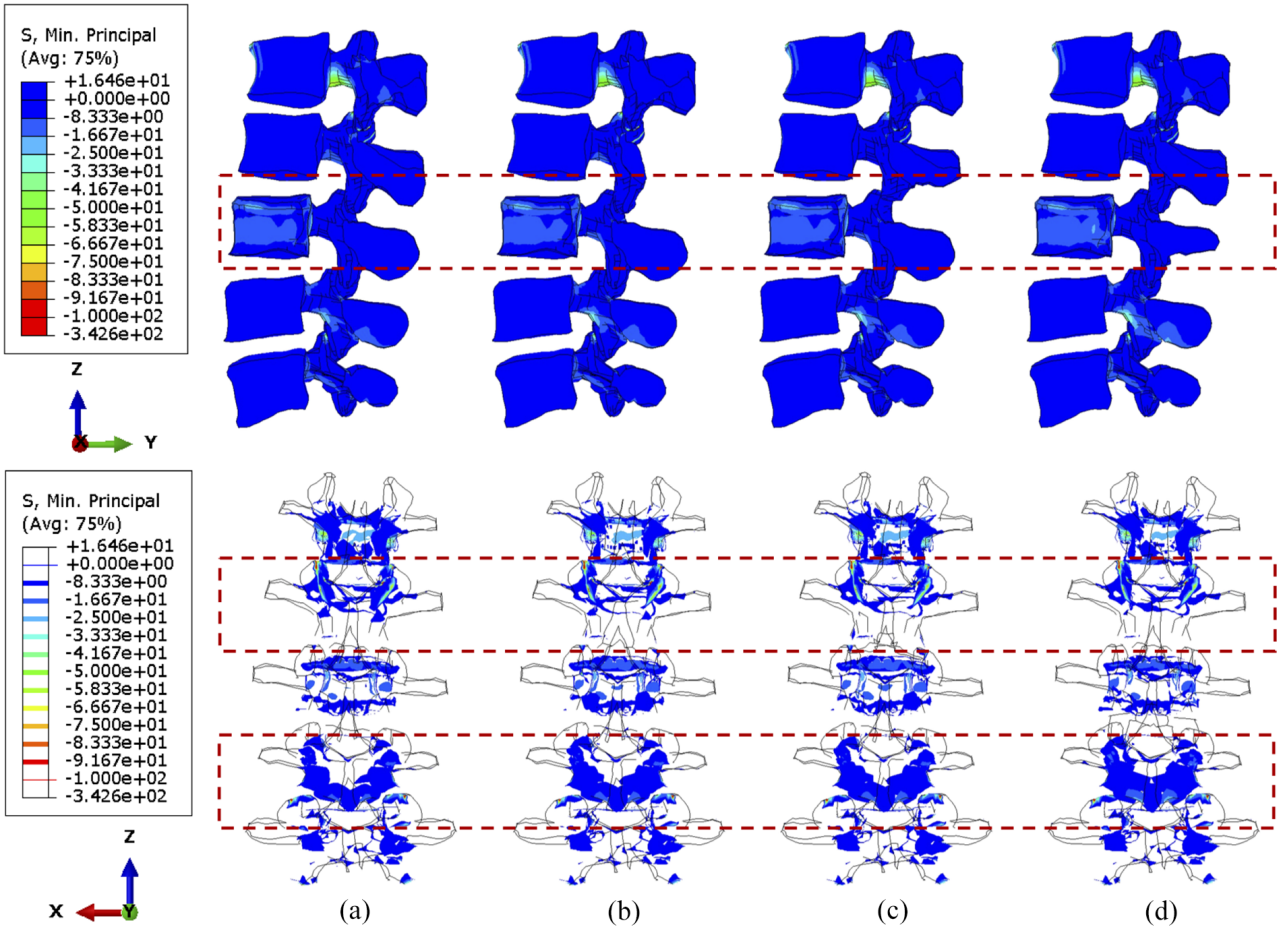
The minimum principal stress distribution of vertebrae, with 92.1% tumour volume, over different stabilisation systems while imposing flexion. The stabilisation is configured: (a) without cross-link and with the cross-link, (b) at the top, (c) in the middle and (d) at the bottom.

No matter the position of the cross-link, flexion causes serious stress concentration in the upper vertebral posterior elements, particularly in L1–L2 joints, as shown in Figures 8 and 9. The overall variations of stress are not considerable, and the cross-link appears to play a trivial role in the stress reduction of the lumbar spine under a constant flexion degree.

**Figure 10.**
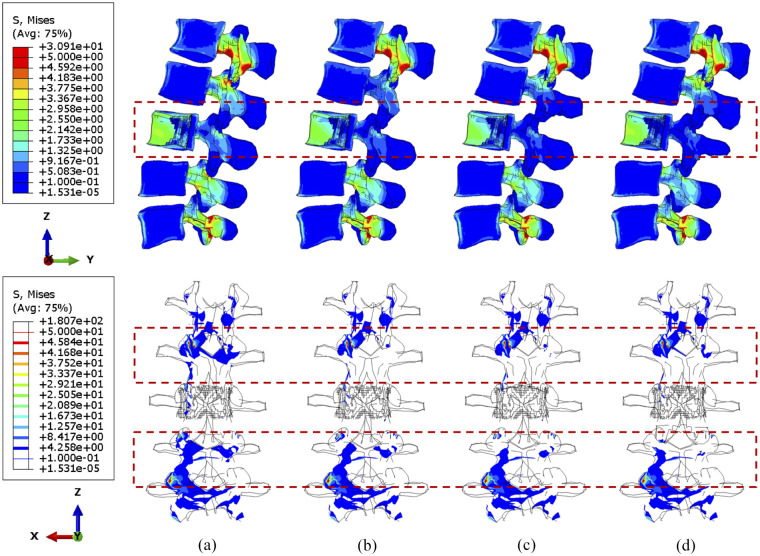
The von Mises stress distribution of vertebrae, with 71.5% tumour volume, over different stabilisation systems under torsion. The stabilisation is configured: (a) without cross-link and with the cross-link, (b) at the top, (c) in the middle and (d) at the bottom.

**Figure 11.**
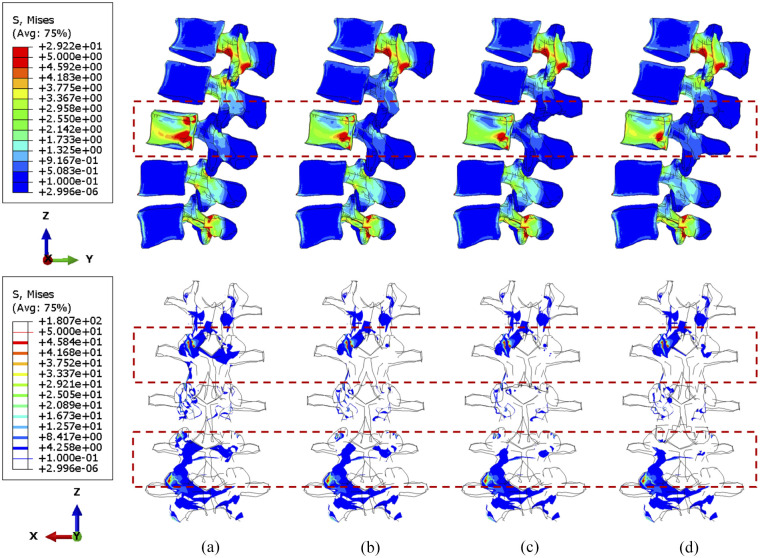
The von Mises stress distribution of vertebrae, with 92.1% tumour volume, over different stabilisation systems under torsion. The stabilisation is configured: (a) without cross-link and with the cross-link, (b) at the top, (c) in the middle and (d) at the bottom.

**Figure 12.**
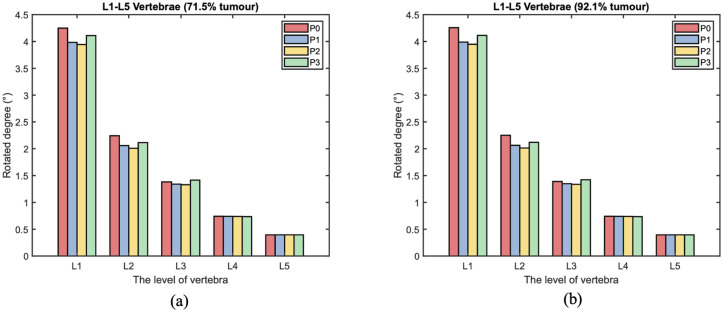
The range of motion (ROM) of each level vertebra over different cross-linked position during torsion. The L3 vertebra is with: (a) 71.5% and (b) 92.1% tumour volume.

During torsion, the von Mises stress mainly concentrates in the posterior elements and the L3 anterior vertebra, as shown in Figures 10 and 11. According to the side view, the cross-link could effectively transfer the shear stress to adjacent vertebrae from the stabilised bony elements. However, the remaining L3 anterior vertebra around the lesion still contains elements with von Mises stress higher than 5 MPa, which is slightly reduced by shifting the cross-link to the top and middle positions. The variations of stress distribution in the posterior elements are observable from the side view, specifically, the stress in the posterior regions is reduced when the cross-link is closer to the level of those regions. Intensive large stress of over 50 MPa only concentrated to L1–L2 and L3–L4 joints, particularly on the left side, which is nevertheless not affected by the tumour size and cross-linked position. In addition, the cross-link positioned at the top provided L1–L3 vertebrae with the most significant resistance of torsion as the range of motion (ROM) are relatively minimal in comparison of the models with four configurations of the stabilisation system, which is consistent in the lumbar spine models with both 71.5% and 92.1% tumour volume (Figure 12).

**Figure 13.**
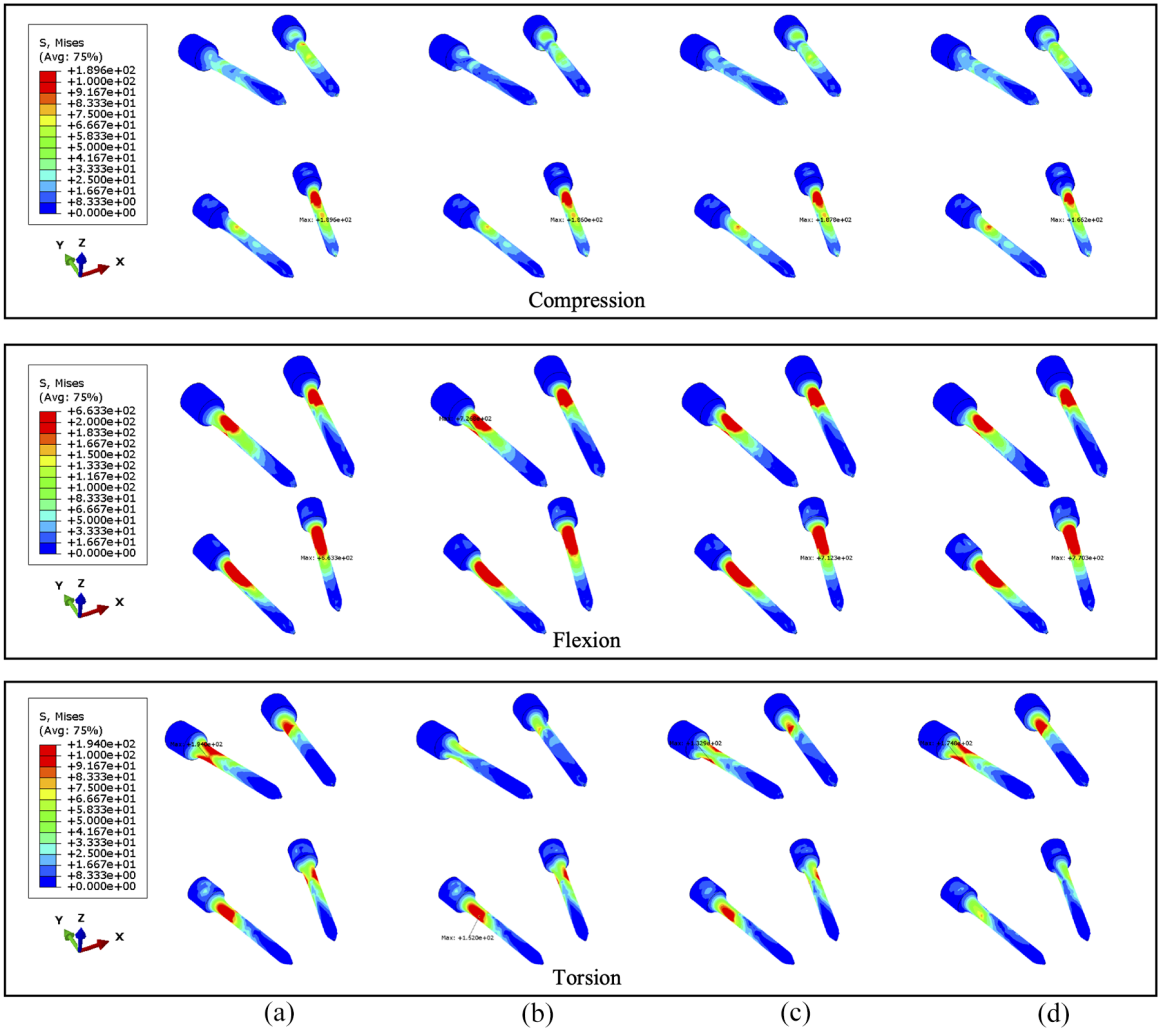
The von Mises stress distribution of screws, with 71.5% tumour volume, under compression, flexion and torsion. The stabilisation is configured: (a) without cross-link and with the cross-link, (b) at the top, (c) in the middle and (d) at the bottom.

**Figure 14.**
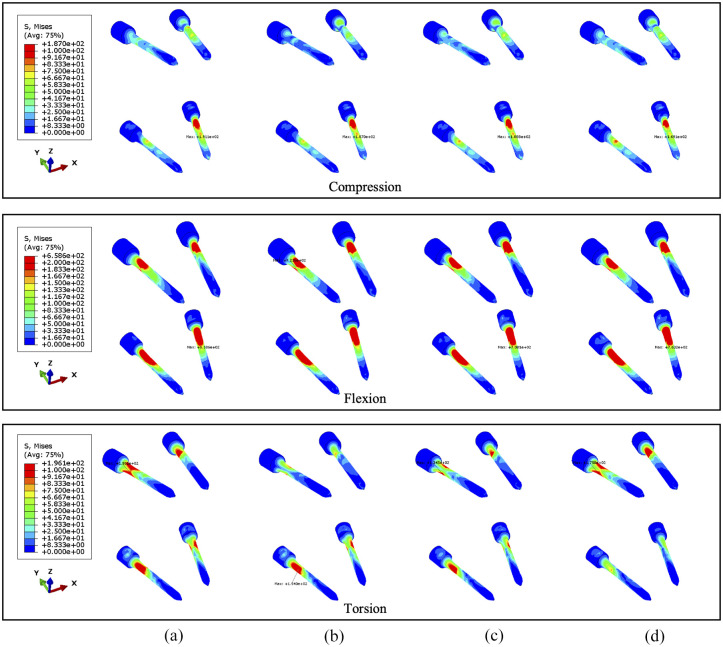
The von Mises stress distribution of screws, with 92.1% tumour volume, under compression, flexion and torsion. The stabilisation is configured: (a) without cross-link and with the cross-link, (b) at the top, (c) in the middle and (d) at the bottom.

Under torsion the screw stress significantly varies with cross-linked positions (Figures 13 and 14). The screw peak stress decreases by around 10% with the cross-link in the lower part and 30% with the cross-link in the middle compared to no cross-link. The stress is often concentrated in the posterior part of the screws and varies significantly depending on the loading. Under flexion or compression, the effect of the position of the cross-link on the screw stress distribution is very small. Tumour size has no significant effect on the stress distribution within the screws. Under torsion, the screw stress is accordingly distributed and reduced when using a cross-link. Interestingly, the similar reduction of stress can be also observed in fused vertebrae when the cross-link is at the same position.

## Discussion

This finite element study aims to investigate potential factors that could affect loading transfer within intact or stabilised vertebrae versus a cross-linked stabilisation system through varying clinical variables including tumour volume and cross-link position. The results indicate that increasing tumour size causes larger stress on the corresponding remaining vertebrae and also affects the contribution of the cross-link stabilisation on the construct. The fixation consistently reduces the stress in the stabilised posterior vertebrae regardless of the tumour growth. Progressive bone loss leads to increasing concentration of the compressive load anteriorly due to the lack of structural competence^53^ resulting from the osteolytic tumour lesion. Since the location of the tumour might not significantly alter the stress distribution within the vertebrae,^35,36,46^ tumour volume could be considered as the primary factor resulting in the mentioned variations of biomechanical behaviours.^54,55^

Similar to compression, the stabilisation contributes to the stress release of a flexed posterior body. Nevertheless, the stress distribution of the anterior vertebrae is unvaried after fixation when the displacement of flexion is constant. In general, the cross-link stabilisation could provide additional structural stiffness for the stabilised vertebrae,^13,22–26^ and the maximum ROM is limited by the fixation device. Accordingly, the fixed vertebrae with stronger stiffness might be subjected to larger stress as well as the reaction moment for the same flexion displacement, which is more considerable with a larger tumour. Significant reaction moment (over 40 Nm) of flexion is found (Figure S4) and it might result from the artefacts of ignoring muscles, as active muscles can resist large flexion moment.^56,57^ However, the trend of increase in the reaction moment of the spine due to stabilisation is consistent with the study by Alizadeh et al.^25^ In terms of the stress distribution, the fixation device consistently has no distinguishable effect on the stress distribution of the anterior body. The risk of vertebral fracture increases more than eightfold when the tumour volume exceeds 30% of the vertebral body,^35^ which suggests that increasing tumour volume could ultimately increase the pressure on the vertebra. Despite the consensus that cross-link stabilisation contributes to stabilisation of the spine in clinical surgery, the device might not be able to effectively decrease pressure within anterior fused vertebrae where a larger tumour volume (over 38.3%) exists.

In addition to the reduction of compressive stress, the cross-link stabilisation improves the resistance of torsion in stabilised vertebrae. The von Mises stress in the vertebrae under torsion was small in comparison to compression and flexion, even if the tumour size increases to 92.1%. Specifically, the cross-link could effectively limit the ROM^25,58,59^ in the axial plane where the spine rotates so that the shear stress can be well distributed into the titanium device from the metastatic vertebrae.

The sensitivity of an instrumented spinal segment to the cross-link position was examined with two sizes of tumour during compression, flexion and torsion. The stress in anterior vertebrae is unvaried against three cross-linked locations, which is contrary to stabilised posterior bodies as most of the load is transferred through the posterior elements. In addition, the cross-link at a lower level brings less structural stability to upper stabilised vertebrae (L2–L3), hence the L3 anterior vertebra is under additional but not significantly large pressure in the models with the cross-link at the bottom. In turn, the relatively higher stress concentrated on adjacent joints and posterior elements, and the stress is not symmetrically distributed between different sides of the spine under uniform compressive loadings. The asymmetricity of the biomechanical behaviours could be explained by the anatomical structure and alignment of the facet joints,^33^ the consequence of which might be potential fatigue and wear on one side of the facets, and clinically relevant amounts of pain.

With respect to the specific numerical results of screws, the considerable reduction of screw stress can be seen under torsion when the cross-link is in the middle due to its positive role in the stability during rotational motions.^60^ Whilst previous study indicates the cross-link related stress reduction of the screw neck during compression and torsion,^25^ there is no benefit to the stabilised spine under a constant flexion degree, which could be a potential risk for the failure of instrumented surgery due to the possibility of fatigue rod fracture.^61^ The addition of a cross-link could increase the stiffness of the stabilisation in the sagittal plane so as to prevent the lumbar spine from larger deformation under the same compressive and shear force. A finite element study^25^ illustrates that the shapes of cross-link do not have a significant impact on the spinal stability and stiffness in short fused segments (L2–L4). Nonetheless, two studies^25,58^ both showed that the stress on the screws decreases by nearly 20% while mounting the horizontal cross-link under axial rotation, which is consistent with results presented in this study. Notably, Wang et al.^58^ suggests that the optimised location of the cross-link should be at upper vertebral level due to larger stress on proximal segments. Likewise, evidence has been building that the cross-link could provide the maximum stability when mounting it more proximally nearer to the top of the rods,^31,32^ which is consistent with our results. Despite the slightly better performance of the cross-link close to L4 vertebra under compressive loads, the stability of the stabilised spine would be reduced so as to destabilise the upper vertebrae (L2–L3) during torsion. As a consequence, the structural stability and stiffness of the stabilised lumbar spine dissimilarly reflect the stress of vertebrae under different loadings. Considering the critical factors of fracture risk,^35,45,46^ the setup of the cross-link located at L2 or L3 could maximise the effects of the stabilisation system on transferring large stress and improve the stability of the stabilised spine. Moreover, the probability of postoperative breakage on pedicle screws may be reduced by mounting the cross-link in the middle of fused vertebrae due to more uniform stress.

This study has several limitations. For instance, the tumour lesion is defined as a porous and inhomogeneous substance,^45^ which could be described by strain energy potentials instead of orthotropic elastic property assigned in this constitutive model. In addition, the study was carried out with constant loadings applied to computational models. However, there might be differences in the stress distribution of the lumbar spine under various rates of incremental loadings.^35^ In fact, the compression loading could be maximised to over 1000 N,^21^ but the lower compressive force represents the gravity of the upper trunk and a representative loading condition to control the compressive force to 1000 N in this study. Accordingly, the critical stress of vertebral fracture was not examined in this study, instead the variations of vertebral stress were presented and analysed. Despite detailed results for sensitivity study, horizontal comparison can enhance the credibility of the finite element analysis, particularly for ROM of each vertebra (Figure 12) due to the lack of corresponding stress analysis in previous research. Intradiscal pressure (IDP) from the patient-specific model without tumour under compression of 300 and 1000 N is compared to experimental data^62^ in Figure S5. The IDP from each level of disc is in the reported range (0.5–2.3 MPa) of a in vivo study^63^ and close to the in vitro experiment^62^ as well as other validated FE models,^64^ whereas the magnitude of IDP from each level of disc is higher in FE models from this study. Different measurements, such as entire lumbar spine^65^ and isolated adjacent segments,^66^ could lead to significant difference in IDP,^67^ hence the selection of experimental is also important for assessing model credibility. More precise validation can be conducted for prediction of lumbar spine biomechanics after surgery in the future.

## Conclusions

This study analysed the effects of tumour size on intact and stabilised lumbar spines through specialised FE models. The effectiveness of the cross-link was examined by differentiating its position within the stabilisation system. The cross-link stabilisation could effectively release the resultant pressure within the posterior bodies. However, the tumour volume is negatively associated with the effects of the fixation device on stress reduction of L3 anterior vertebra under compressive loads. Due to the cross-link, the negative impact does not appear in the rotated lumbar spine models and the shear stress significantly decreases in stabilised vertebral segments, though a growing tumour persistently results in larger concentrated stress in the eroded anterior vertebra. Likewise, the stabilised spine is more sensitive to the positions of the cross-link during torsion compared to compression and flexion. The cross-linked positions are not sensitive to compressive and flexed loadings. The variations of the stress reduction could be observed in both vertebrae and screws when varying the cross-linked position, therein the cross-link in the middle effectively reduces the concentration of stress on the screw neck. Overall, the cross-link is necessary for a lumbar spine with a tumour, and careful consideration when altering the position of the cross-link can not only release the shear stress but also assist the cancer treatments. Further examination on the critical stress and stability of the stabilised lumbar spine is needed so that clinical instrumented stabilisation surgery could be specifically optimised.

## Supplemental Material

sj-docx-1-pih-10.1177_09544119251348279 – Supplemental material for A finite element study of the effect of cross-link stabilisation in a lumbar spine tumour model Supplemental material, sj-docx-1-pih-10.1177_09544119251348279 for A finite element study of the effect of cross-link stabilisation in a lumbar spine tumour model by Juntong Lai, James Tomlinson, Lee Breakwell and Damien Lacroix in Proceedings of the Institution of Mechanical Engineers, Part H: Journal of Engineering in Medicine

sj-jpg-6-pih-10.1177_09544119251348279 – Supplemental material for A finite element study of the effect of cross-link stabilisation in a lumbar spine tumour model Supplemental material, sj-jpg-6-pih-10.1177_09544119251348279 for A finite element study of the effect of cross-link stabilisation in a lumbar spine tumour model by Juntong Lai, James Tomlinson, Lee Breakwell and Damien Lacroix in Proceedings of the Institution of Mechanical Engineers, Part H: Journal of Engineering in Medicine

sj-tiff-2-pih-10.1177_09544119251348279 – Supplemental material for A finite element study of the effect of cross-link stabilisation in a lumbar spine tumour model Supplemental material, sj-tiff-2-pih-10.1177_09544119251348279 for A finite element study of the effect of cross-link stabilisation in a lumbar spine tumour model by Juntong Lai, James Tomlinson, Lee Breakwell and Damien Lacroix in Proceedings of the Institution of Mechanical Engineers, Part H: Journal of Engineering in Medicine

sj-tiff-3-pih-10.1177_09544119251348279 – Supplemental material for A finite element study of the effect of cross-link stabilisation in a lumbar spine tumour model Supplemental material, sj-tiff-3-pih-10.1177_09544119251348279 for A finite element study of the effect of cross-link stabilisation in a lumbar spine tumour model by Juntong Lai, James Tomlinson, Lee Breakwell and Damien Lacroix in Proceedings of the Institution of Mechanical Engineers, Part H: Journal of Engineering in Medicine

sj-tiff-4-pih-10.1177_09544119251348279 – Supplemental material for A finite element study of the effect of cross-link stabilisation in a lumbar spine tumour model Supplemental material, sj-tiff-4-pih-10.1177_09544119251348279 for A finite element study of the effect of cross-link stabilisation in a lumbar spine tumour model by Juntong Lai, James Tomlinson, Lee Breakwell and Damien Lacroix in Proceedings of the Institution of Mechanical Engineers, Part H: Journal of Engineering in Medicine

sj-tiff-5-pih-10.1177_09544119251348279 – Supplemental material for A finite element study of the effect of cross-link stabilisation in a lumbar spine tumour model Supplemental material, sj-tiff-5-pih-10.1177_09544119251348279 for A finite element study of the effect of cross-link stabilisation in a lumbar spine tumour model by Juntong Lai, James Tomlinson, Lee Breakwell and Damien Lacroix in Proceedings of the Institution of Mechanical Engineers, Part H: Journal of Engineering in Medicine
