# Supplementary material for: A finite element study of the effect of cross-link stabilisation in a lumbar spine tumour model
Source: Proc Inst Mech Eng H. 2025 Jul 7;239(7):607–23. doi: 10.1177/09544119251348279 (PMC12287565; doi:10.1177/09544119251348279)
Supplement: sj-docx-1-pih-10.1177_09544119251348279 – Supplemental material for A finite element study of the effect of cross-link stabilisation in a lumbar spine tumour model [file sj-docx-1-pih-10.1177_09544119251348279.docx]

**Supplemental material**

Fig S1. The classification of material properties with regard to the ligaments within an FSU.

Fig S2. The classification of material properties with regard to the intervertebral disc.

Fig S3. The classification of material properties with regard to the vertebra.

Fig S4. The reaction moment of the intact and fused lumbar spine tumour models during 10º flexion.

Fig S5. The comparison of intradiscal pressure (IDP) at L1-L5 between the lumbar spine model without tumour lesion and in vitro data from Brinckmann & Grootenboer. Mean values of IDP from experiments are presented on the right side with the error bar indicating the standard deviation.
