# Supplementary figures and images for: A finite element study of the effect of cross-link stabilisation in a lumbar spine tumour model
Source: Proc Inst Mech Eng H. 2025 Jul 7;239(7):607–23. doi: 10.1177/09544119251348279 (PMC12287565; doi:10.1177/09544119251348279)

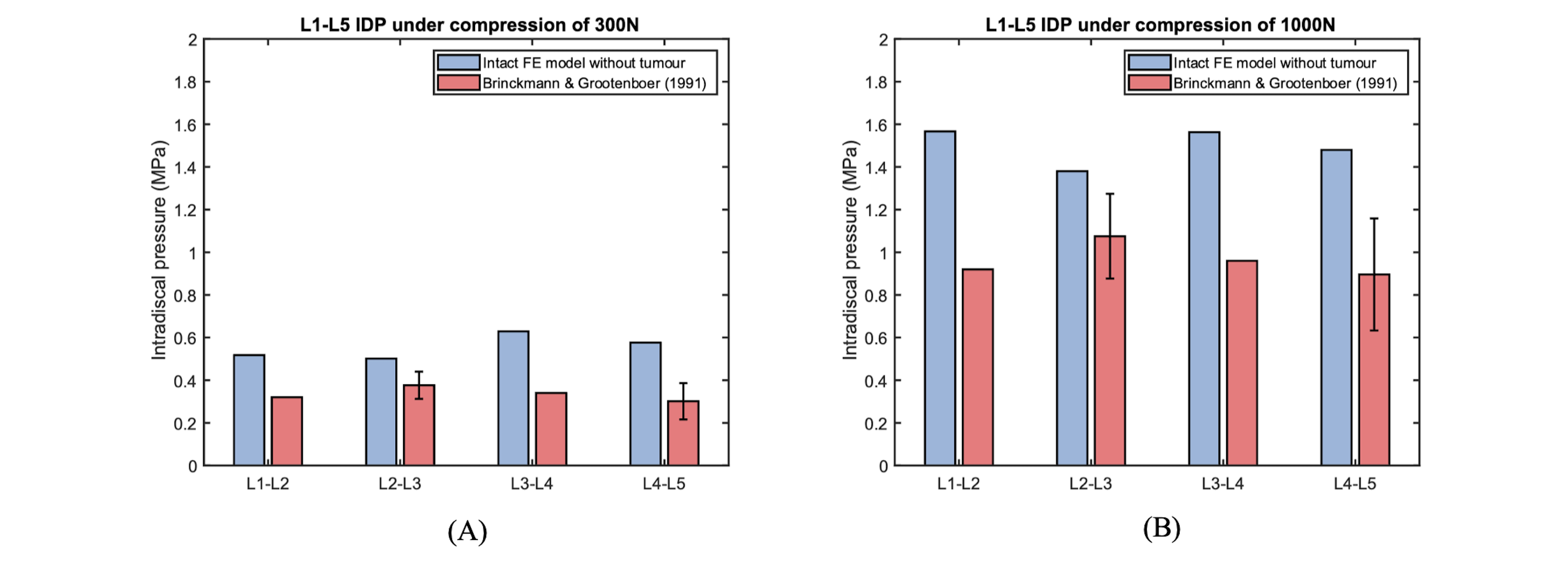

Supplement: sj-jpg-6-pih-10.1177_09544119251348279 – Supplemental material for A finite element study of the effect of cross-link stabilisation in a lumbar spine tumour model [file sj-jpg-6-pih-10.1177_09544119251348279.jpg]

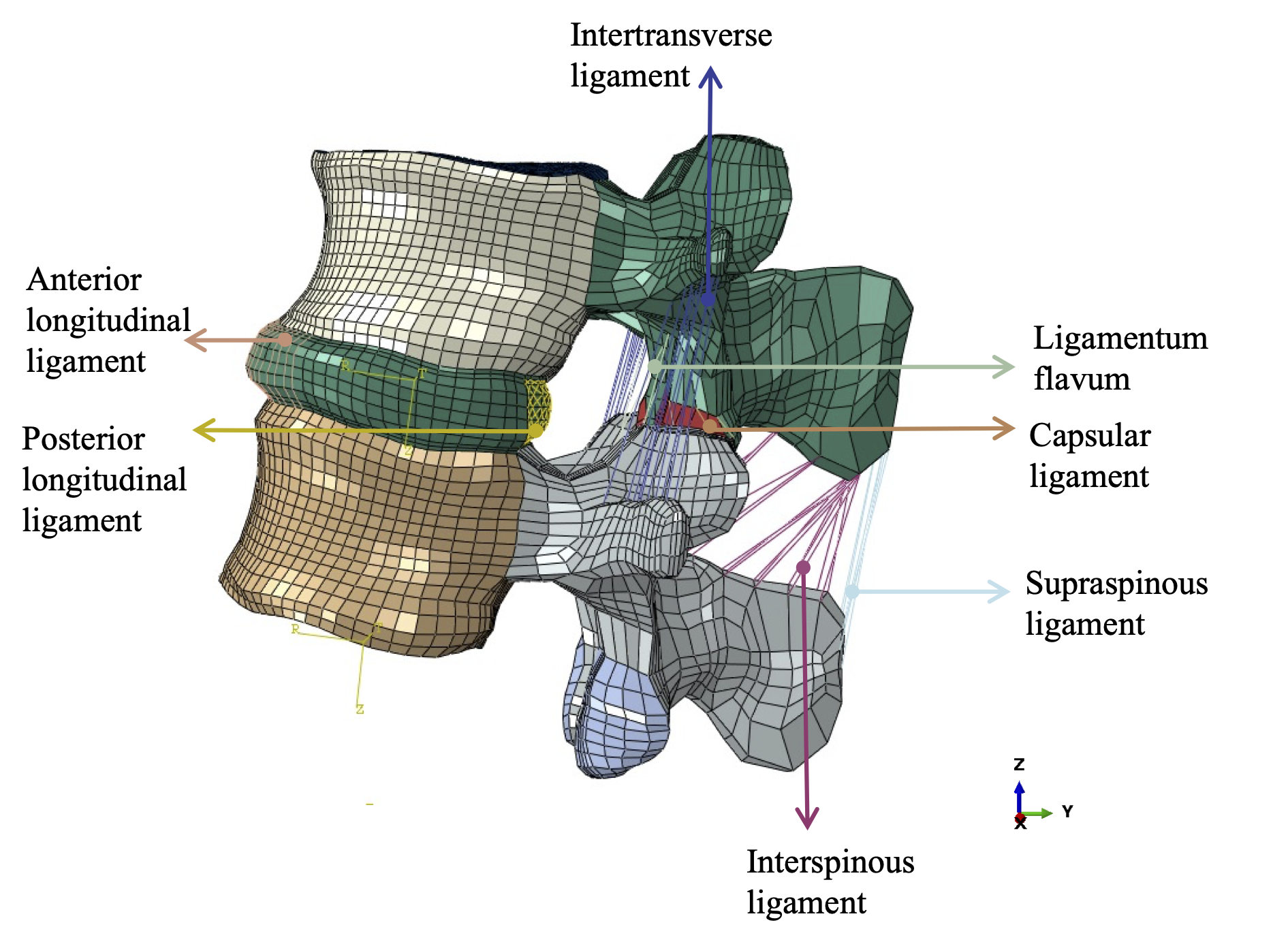

Supplement: sj-tiff-2-pih-10.1177_09544119251348279 – Supplemental material for A finite element study of the effect of cross-link stabilisation in a lumbar spine tumour model [file sj-tiff-2-pih-10.1177_09544119251348279.tiff]

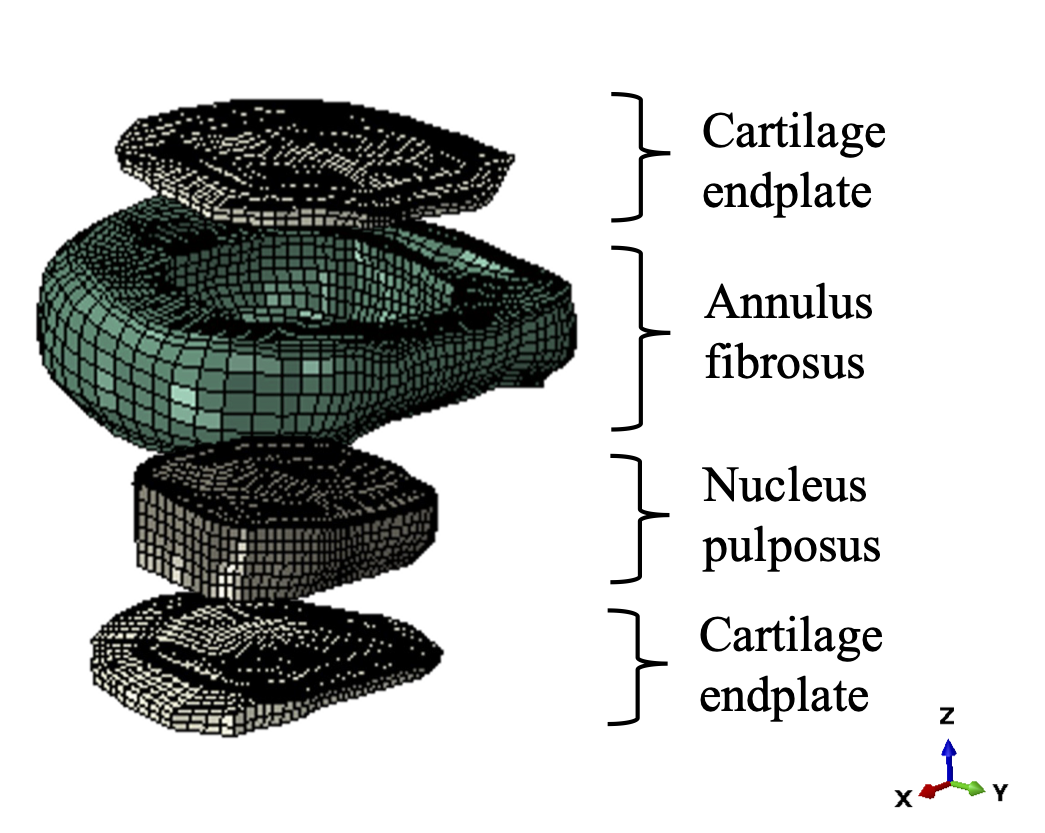

Supplement: sj-tiff-3-pih-10.1177_09544119251348279 – Supplemental material for A finite element study of the effect of cross-link stabilisation in a lumbar spine tumour model [file sj-tiff-3-pih-10.1177_09544119251348279.tiff]

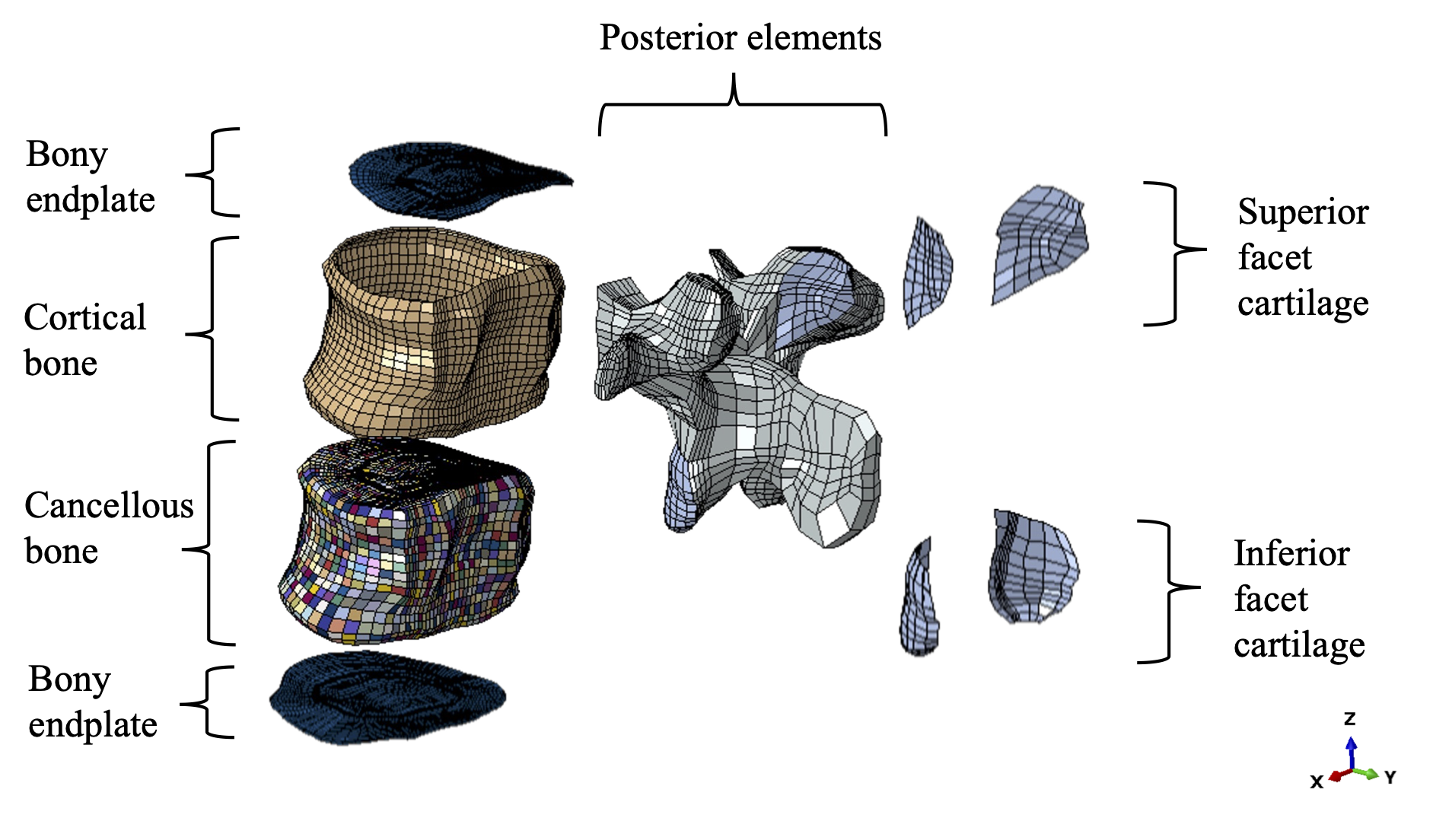

Supplement: sj-tiff-4-pih-10.1177_09544119251348279 – Supplemental material for A finite element study of the effect of cross-link stabilisation in a lumbar spine tumour model [file sj-tiff-4-pih-10.1177_09544119251348279.tiff]

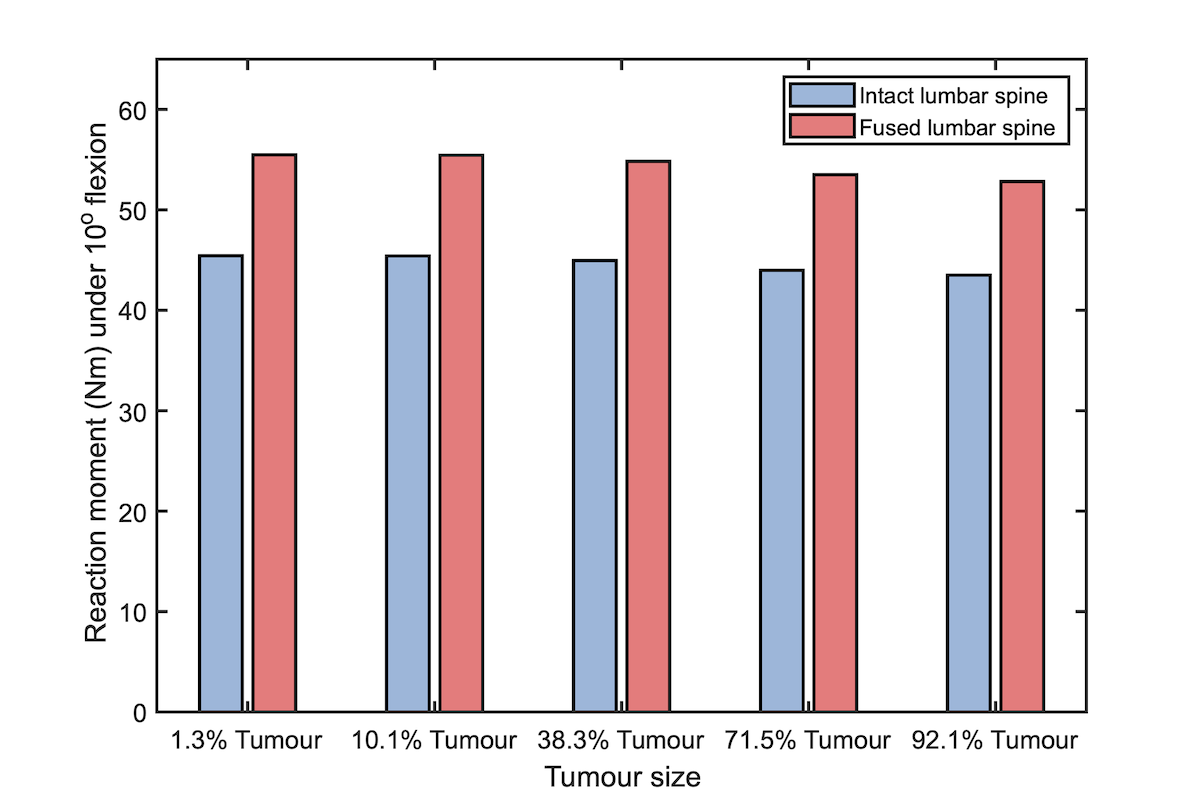

Supplement: sj-tiff-5-pih-10.1177_09544119251348279 – Supplemental material for A finite element study of the effect of cross-link stabilisation in a lumbar spine tumour model [file sj-tiff-5-pih-10.1177_09544119251348279.tiff]
